# LinguAPP: An m-Health Application for Teledentistry Diagnostics

**DOI:** 10.3390/ijerph19020822

**Published:** 2022-01-12

**Authors:** Matia Fazio, Christian Lombardo, Giuseppe Marino, Anand Marya, Pietro Messina, Giuseppe Alessandro Scardina, Antonino Tocco, Francesco Torregrossa, Cesare Valenti

**Affiliations:** 1 Department of Mathematics and Informatics, University of Palermo, 90123 Palermo, Italy; matia.fazio@gmail.com (M.F.); peppe98.marino@gmail.com (G.M.); entonytocco@gmail.com (A.T.); 2 Department of Surgical Oncological and Stomatological Disciplines, University of Palermo, 90133 Palermo, Italy; christianlombardo778@gmail.com (C.L.); pietro.messina01@unipa.it (P.M.); 3 Center for Transdisciplinary Research, Saveetha Dental College, Saveetha Institute of Medical and Technical Science, Saveetha University, Chennai 600077, India; amarya@puthisastra.edu.kh; 4 Department of Orthodontics, Faculty of Dentistry, University of Puthisastra, Phnom Penh 12211, Cambodia; 5 Department of Informatics, King’s College London, London WC2R 2LS, UK; d.ftorregrossa@gmail.com

**Keywords:** dentistry diagnostics, m-health application, medical questionnaire, teledentistry

## Abstract

An Android/iOS application for low-cost mobile devices to aid in dental diagnosis through questionnaire and photos is presented in this paper. The main purposes of our app lie in the ease of use even for nonexperienced users, in the limited hardware requirements that allow a wide diffusion, and in the possibility to modify the questionnaire for different pathologies. This tool was developed in about a month at the beginning of the COVID-19 (SARS-CoV-2) pandemic and is still in use in Italy to allow support to patients without going to the hospital, if not strictly necessary.

## 1. Introduction

Recently, m-health applications (i.e., medicine supported by mobile devices) have been applied to various medical fields due to the wide diffusion of mobile devices, the low cost of Internet connection, and the ability to reach substantially almost any remote area [1,2]. Systems to perform an initial diagnosis automatically have been presented in the literature, but a comparison with a medical specialist who provides the final indications and therapy is always appropriate [3,4]. Machine learning for the triage of skin lesions was proposed in [5], while artificial intelligence for ophthalmic screening is described in a broad sense in [6]. Sometimes the use of additional hardware is required to enhance the optics or lighten the computational load on the mobile device [7,8,9,10,11]. A general review of a variety of m-health applications for chronic conditions and/or diseases is given in [12], while COVID-19 (SARS-CoV-2) teledentistry opportunities are described in [13,14].

During the early stages of the current COVID-19 pandemic condition, we needed to develop, in a very tight time constraint, a mobile device app to provide medical staff with useful dental information. A further fundamental requirement was the need not to use any additional hardware and to take full advantage of the features of entry-level cell phones. This allowed to give immediate assistance to the population and to arrange an eventual dedicated hospital intervention, avoiding dangerous contact with the rest of the hospitalized patients. Our app, called LinguApp, is freely downloadable for Android and iOS operating systems and is still in use at the Department of Surgical Oncological and Stomatological Disciplines in Palermo, Italy, even though it provides support for the entire country of Italy. This tool submits a triage questionnaire, formulated in a simple way for nonspecialists, and prompts them to take at least two photos to highlight lesions within their mouth without the use of any template to refer. The specialist is then contacted through an e-mail and can provide a preliminary diagnosis by means of a web interface. One of the main advantages of this application lies in the simplicity with which the questionnaire can be managed through the same interface. This feature allows LinguApp to be modified so that after the COVID-19 emergency, it is aimed directly at medical specialists with a more complete and unfamiliar questionnaire, translated into different languages and used in different contexts that can benefit from a questionnaire with attached photos and videos.

In the field of oral pathologies, it is fundamental to make a diagnosis [15,16]: through (*dia*) knowledge (*gnosis*), we aim to answer the question: What is the patient suffering from? In order to have enough knowledge to allow to name a wound, it is necessary to understand the visited patient: lifestyle habits, assumption of drugs, presence of systemic pathologies (generalized or localized), familiarity for certain medical conditions, history of the lesion in the matter, related markers, and symptoms are all aspects that will have to be collected and connected to each other to allow a diagnostic suspicion [17]. This suspicion will be confirmed with the help of any instrumental/histological/microbiological/hematological/clinical examinations [18,19,20]. Consequently, the diagnosis requires the acquisition of certain information essential for the understanding of the clinical condition of the patient.

The objective of this study is the development of a mobile application to gather the first piece of information for the diagnosis. This application does not have any diagnostic confirmation purpose, which remains exclusively for the objective and instrumental examination in presence, but allows to start a diagnostic path in line with the indications and directives during the quarantine due to COVID-19. Considering the high risk of viral contagion by airborne propagation that dental procedures have inherent in their specificity, these organizations suggest to limit all dental examinations in presence with the exception of urgency and absolute non-deference. During the first pandemic period (in Italy, approximately in March and April 2019), there was the deontological indication to limit treatments only in the case of urgent and unavoidable emergencies, suggesting remote consultation, video consulting, and specialist advice by phone. By using LinguApp on any Android or iOS mobile device, the patient describes his/her symptoms, takes some pictures, and sends all the data directly to a team of specialists who evaluate the actual need to refer the patient to highly specialized centers. Our application is intended mainly for immobilized and noncooperative patients in difficult locations who cannot go to medical facilities during this emergency period [17].

The ability to distinguish one lesion from another is not always so simple, nor so fast. We designed an interactive procedure to navigate a directed graph that helps us identify various lesions hypothetically detectable, in relation to the soft tissues of the oral cavity. The app imposes an order of questions to be answered unambiguously: in this way, the questions divide the large group of “mouth lesions” into smaller, different groups, allowing us to reach a diagnosis of presumption by evaluating the attached photos.

## 2. Methods

To solve the problem, among the possible viable implementations, the choice fell on the development of two distinct products: a mobile application aimed at patients, and a dashboard or content management system aimed at clinicians.

The choice, on the patients’ side, was dictated by the widespread diffusion that smartphones have had in recent years, due to their typically low cost and ease of use that result from their simplified interfaces. This is perfectly in line with the features required by our service, and we have therefore developed it to make it accessible even by patients who are not experienced with technology, through the use of standard procedures that they are already familiar with.

As for the activities reserved for the clinicians, we have taken a different approach. An additional section within the same application, designed to process requests and only available to the medical staff, could have been a viable option. However, we considered this to be impractical and instead chose to develop a dedicated system, accessible through a website, in which we sacrificed the ease of use in favor of a more comprehensive tool that could also be used on desktop computers.

**Figure 1:**
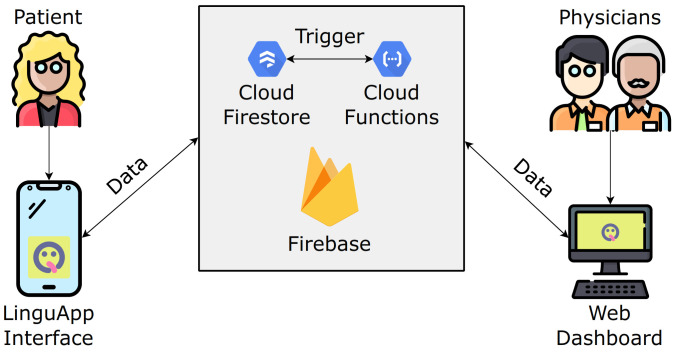
Simple sketch of the proposed methodology.

These two pieces of software, although independent, still need to communicate with each other. To this end, we used the Firebase platform offered by Google, which provides an infrastructure for the development of mobile and web applications, comprising a number of services such as hosting, databases, patient authentication, and a simplified management of push and email notifications [21]. A sketch of the whole architecture is presented in Figure 1.

The development of smartphone applications is a process that normally follows two parallel paths, for Android and iOS devices, respectively. These two systems are fundamentally different from one another, and each has dedicated development tools. However, often developers do not need to use any platform-specific tool or feature, or even differentiate their products for the two operating systems. This holds true in our case as well, where speed of development was crucial to achieve the goal. For this reason, we opted for a hybrid development system that would allow simultaneous development on both operating systems. We chose React Native [22], an open-source framework made by Facebook to create mobile applications in JavaScript. Similarly, and for consistency, the dashboard was built using its counterpart for web applications, React [23].

### 2.1. Decision Graph

The primary purpose of our application is to collect information about patients and their symptoms using a well-structured approach. This was accomplished by asking the patient to perform a self-inspection by choosing, from a set of fixed alternatives, the options that best fit their problem. We considered the possibility of allowing patients to openly describe their symptoms, but this could have led to inaccurate or incomplete information that would have required subsequent refinements. Instead, we wanted an exhaustive application in providing data and alternatives to the patient, starting from the first interaction. This observation led to the issue of properly structuring the progression between questions.

At first, we considered using a decision tree, but this turned out to be insufficiently flexible for our needs. This is because, in the considered survey, some of the questions are repeated in distinct paths and the use of a decision tree would result in unnecessary redundancies. Indeed, when using such a structure, a question may only be preceded by a specific series of answers, instead of several distinct series.

**Figure 2:**
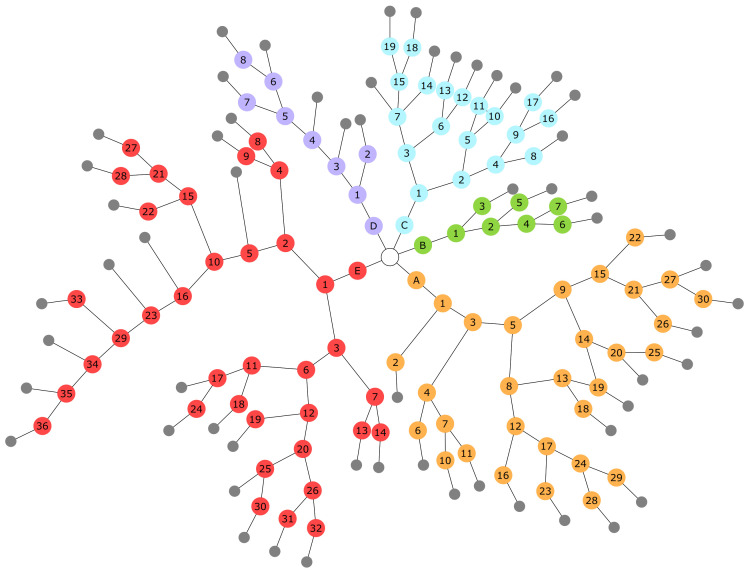
Graphical representation of the questionnaire. Colours indicate the five main branches, corresponding to the possible answers of the first query. Further details are provided in the Additional Material (Appendix A). The reader is referred to the electronic version of the article for interpretation of the colours in this figure.

For this reason, we opted for an acyclic directed graph where each node represents a question, and, as such, contains a set of strings. In particular, each alternative is indicated by a string that can help in making a diagnosis (e.g., description of symptoms, presence of pre-existing conditions, lifestyle habits). Though not required, a node may also contain a header string that provides further details related to all alternatives. Each alternative is also associated with a reference to the next question, which can be interpreted as an edge in the graph (Figure 2). The keys of the JSON code, provided in English as Additional Material (see Appendix A), are the labels of the individual nodes. The first question, common to all subgraphs, is proposed to the patient and indicated here by the white circle; the small disks in gray represent the invitation to take photos. LinguApp notifies the specialists’ team via email that a new request was added so that they can quickly assess the case and get in touch with the patient. Due to privacy reasons, no sensitive data is attached in the email.

Once we established the data structure, we needed to find a suitable way to represent and store it. We opted for the JSON format, as it is highly flexible and natively handled by both the JavaScript language and the Firebase’s database service, called Firestore. Initially, one might think of nesting a node inside its ancestor, following the typical structure of a JSON file, but this is not adequate for our data structure, as, once again, it would describe a tree. The solution we have employed requires all nodes be stored at the same level and that each be associated with an identifying key. Our JSON representation can be examined in the Additional Material (Appendix A).

It is possible to represent the path that a patient follows through the graph by means of a list in which the keys of the visited nodes are stored. An algorithm for visualizing a path is implemented in both the mobile and web application and follows the pseudocode in Appendix A, Algorithm A1.

### 2.2. Firebase Backend

Firestore is the Firebase component that manages databases; it is structured in collections and each of them contains documents, which represent a set of key-value pairs. The most relevant collections for the functioning of our software are those that contain the surveys and the paths taken by the patients.

Each survey was mapped to a graph represented in JSON, as described in the previous section, and stored in a document within the same collection. The contents are maintained by system administrators, who have the option of adding new surveys and new questions in an existing one, though deletions cannot be made, as it would no longer be possible to interpret the paths previously taken by patients (i.e., we impose that our application is backward compatible).

The collection of paths contains one document for each path. The status for a path can be one of “draft”, “awaiting photos”, “awaiting feedback”, and “completed”. A path contains a reference to the patient who created it, the list of choices, an indication of the status and links to the associated photos, which are stored through the storage service. For security and privacy reasons, only administrators have access to the whole documents. When a patient completes the self-inspection, including uploading photos, members of the medical staff are notified via email, leveraging Firebase’s cloud functions. Similarly, when clinicians provide feedback, the patient involved is notified via push notifications and email.

This service is available upon a sign-up procedure, which is structured in two steps. At first, an account is created through an authentication service (email, Google, Apple ID) and only afterwards do patients provide their personal data and a phone number that will be stored in a dedicated collection. The data entered is validated using other Firebase services.

Moreover, Firebase provides the Remote Config service, which allows administrators to quickly edit text and settings within the application without having to release an update on the stores: LinguApp will download new values at each startup, while the dashboard will update them every time the webpage is refreshed.

### 2.3. Mobile Application

**Figure 3:**
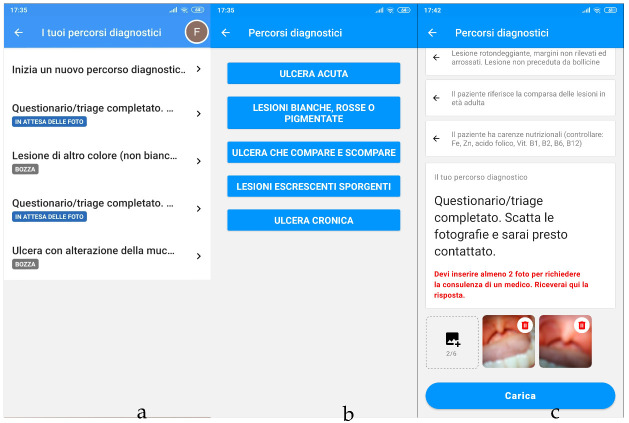
Requests started by a user with their relative statuses color-coded, e.g., “draft” in grey and “waiting for photos” in blue (**a**). First choice of a path (**b**). Request awaiting submission, with all choices already taken and showing the interface for adding photos (**c**). LinguApp features Italian phrases that can be translated quite easily (please see Additional Material).

When first launching LinguApp, the patient must sign up or log in using a previously created account. The first screen shows a short tutorial, after which the patient can start a new path or view a previously created one (Figure 3a).

During the creation of a new path, the patient is asked to select the survey category they consider most suitable among the available ones (Figure 3b). From this point on, the list of decisions made by the patient is treated as a stack that initially contains the key of the first node of the selected category. Any choice made by the patient results in its immediate synchronization on Firestore.

The screen that follows provides a dedicated interface for viewing and interacting with the decisions currently taken, allowing the patient to either advance by answering a new question, or go back to change a previously proposed answer (Figure 3c). The path is shown as a list, where each node corresponds to a panel. The list can be displayed in full or in a compact form: if an answer has yet to be provided, all of its alternatives are shown, whereas if an answer has already been given, it will be the only one displayed. This means that, by default, only one node at a time is fully displayed, namely the last one in the path. However, to allow patients to go back, we made it so that upon tapping a past question, its panel expands again. A question that is fully displayed allows interaction with its alternatives. Selecting one of them will truncate any following given answers and append the key of the node that is pointed to by the selected alternative. Further details on this procedure are provided in Appendix A, Algorithm A2.

When a patient reaches a terminal node, the path changes to “waiting for photos”, and displays the interface for uploading photos. Upon successful submission, the status will change to “waiting for feedback”. When a clinician provides feedback, it is shown on this screen. For the specific problem to solve, we preferred to make the patient shoot photos, but LinguApp can be modified to capture videos as well.

We have done our best to make the app as undemanding as possible in terms of operating system, hardware resources, and memory space. The minimum required version of Android is 4.1, released in 2012; the size to download the app from the store is about 23 megabytes, and after installation it takes about 46 megabytes. In the case of iOS, at least the 9.0 version is required, 24 megabytes have to be downloaded from the store, and just 16 megabytes are occupied on the device. These differences are due to the optimization policies of the respective operating systems.

### 2.4. Web Application

Login to the dashboard, which is run on Firebase’s hosting service, is performed via email and password or via a Google account. The sign-up of clinicians is performed by the system administrators to prevent arbitrary individuals from accessing these functionalities. Although this procedure is cumbersome, developing a more extensive feature would be futile.

The web portal has a dedicated section for content management where it is possible to edit questions and answers. As already mentioned, the backward compatibility with previous completed paths limits clinicians’ freedom of editing surveys. This is a minor limitation, though, as the surveys were designed to remain essentially unaltered.

**Figure 4:**
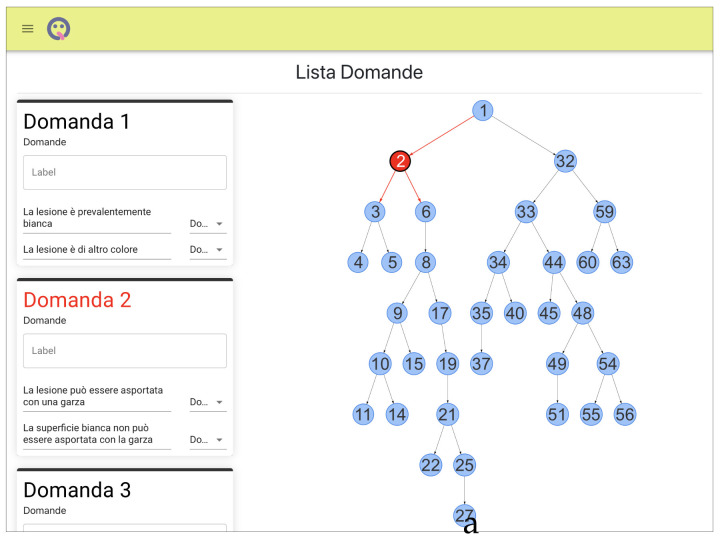

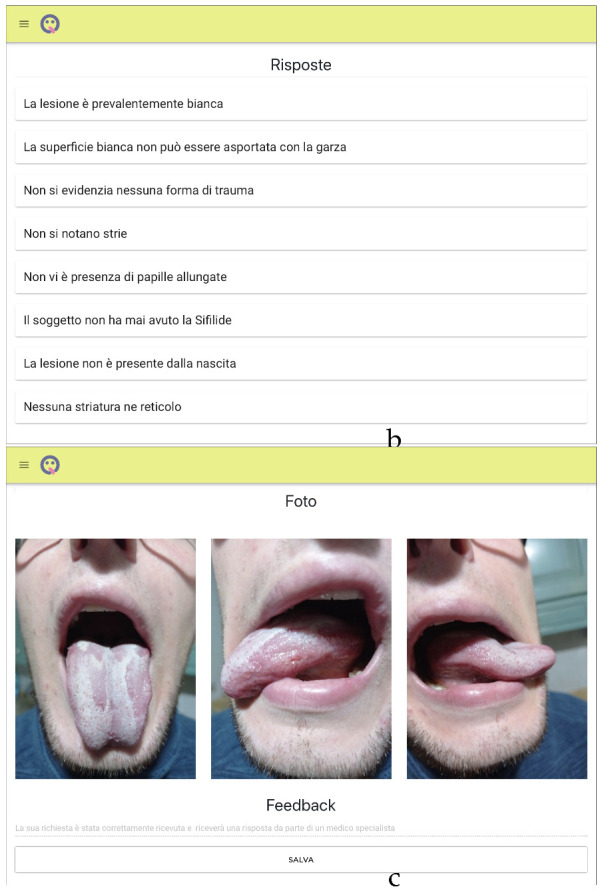
The web interface allows to create and modify the questionnaire, also through graphic representation (**a**). It is possible to delete or view details for an individual request on a dedicated page containing patient contact information, the selections made (**b**), and the attached photos (**c**). Sensitive data has been censored. LinguApp features Italian phrases that can be translated quite easily (please see Additional Material).

The other, and most important, section is the one from which medical staff can review patient submissions. As all clinicians have equal access to this section, being able to distinguish between requests that still need attention and those that have already received feedback is essential for proper and effective cooperation. To achieve this, the completed requests are marked by a label. The details of a path are displayed in a dedicated page that shows the given answers, the photos included, and the patient’s contact details. Once a clinician has formulated a feedback, based on the available information, the patient will be contacted via a dedicated input field on the page or through the contact information (Figure 4).

### 2.5. Privacy and Security

Confidentiality is fundamental in the medical field, so any application that operates in this area must provide strong guarantees on the treatment of user data. Patients that use LinguApp need to provide personal information such as their full name, email address, and phone number. To start a diagnostic path with a clinician, they also need to answer questions that could reveal more sensitive data, and then take pictures of their lesions. Our approach to preserving users’ privacy and security is twofold.

First, we leverage the built-in security provided by all Firebase services, which is equivalent to that of mainstream products. Firebase rejects all requests coming from untrusted clients. Indeed, our clients have been configured for each of the three platforms, iOS, Android, and the web, through the use of private configuration files that guarantee their authority, and all communications are carried out through a private channel.

Second, we add a layer of protection by limiting the access to the resources, particularly to the collection of patients’ data and paths and to their pictures. The access is controlled through a variety of Firebase services. First of all, the sign-up procedure makes use of Auth to ensure that all users, both patients and clinicians, have a personal account, and of Custom Claims to ensure that their account is “activated” before they can access any other functionality. In other words, users have to provide valid information, completing both steps of the sign-up process, before the service becomes available. When an “activated” user makes a request, only the user’s own data will be disclosed. This is guaranteed via Security Rules that are applied in Firestore and Storage before a request is processed.

According to Firebase’s best practices for security and privacy, these restrictions should be enough to ensure that the visibility of information is limited to the account, and by extension the patient, who created it. Indeed, even if a third party were to gain access to the clients’ source code, they would not be able to obtain additional data due to server-side restrictions.

As noted in the Terms and Conditions of the software, all data collected via LinguApp can be accessed by clinicians at Department of Surgical Oncological and Stomatological Disciplines in Palermo, Italy, solely for diagnostic purposes and for health and administrative records required by law. If necessary, data might also be disclosed to a patient’s treating clinician, other healthcare personnel, and dental laboratories. Additionally, in order to ensure European General Data Protection Regulation compliance, patients can download their data and request deletion, should they wish to do so.

## 3. Results and Discussion

LinguApp has made it possible, and still makes it possible, to manage patients who, for various reasons, require a prodromal remote consultation. On the basis of the anamnestic documentation provided online by the patients and the evaluation of the images sent, the medical team expresses its opinion on a therapeutic indication or on the need for further diagnostic investigations that require an in-person consultation. In this latter case, the appropriate hospital structure to manage the case is indicated [17,18]. During this pandemic period, the app has enabled numerous patients to find a solution to their problem without having to leave their homes. In other cases, the patient had to go to a hospital, but only after the medical team had recognized the real necessity, which is extremely important in order not to unnecessarily burden hospital facilities.

**Figure 5:**
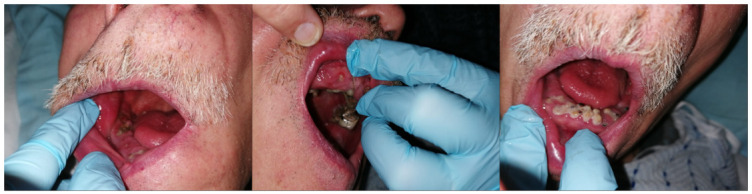
An anonymized real case study. Although no hospital examination was necessary, this example points out the effectiveness of the proposed methodology.

In this case, LinguApp has also enabled carrying out a remote triage which is essential in order to have access to health facilities in Italy, having assessed the low risk of COVID-19 positivity. The app has therefore made it possible to manage some patients at a remote distance but also to accept at the health facility low-risk patients with COVID-19 positivity, thus reducing the risk of patient mobility, which is essential for the containment of the pandemic infection itself. In particular, we believe it is useful to report, among the many requests for assistance, an emblematic case. It regards a disabled and bedridden patient. This consultation request was made by the relatives of the patient who had been experiencing serious difficulties in feeding for several days. From the anamnestic collection it was possible to highlight the frequent use of aerosol therapy containing corticosteroid drugs. The images highlighted the presence of numerous root residues, and the presence of oral extremely erythematous mucosae (Figure 5). Considering the reported semi-liquid diet, the medical team believed that the feeding difficulties could be caused by oral burning, probably related to drug-related mycotic overinfection and also related to the poor oral hygiene conditions due to the general health conditions of the patient. Therefore, it was recommended to follow a topical antifungal therapy for 15 days and also an improvement of oral hygiene conditions through the use of gauzes and a basifying agent based on water and bicarbonate. After 15 days, the patient reported the disappearance of any difficulty to eat and the reduction of the erythema of the oral mucosa.

We think this case absolutely emblematic: indeed, the presence of the disabled patient in a hospital would have involved obvious difficulties, for example, his transport via dedicated means such as an ambulance. Moreover, it would have required the participation of several accompanying persons. The risk to which this patient would have been exposed with numerous comorbidities in case of COVID-19 infection would have placed his life in serious danger, as well as his family members.

## 4. Conclusions

The search for pathological formations or dysembryogenic alterations that can affect the soft tissues of the mouth, either as the only organ in which they are found, or as expression of pathologies that also occur in other sites, shows how many lesions clinically resemble each other [17,18,19,20]. The similarity among the appearance of many lesions represents a difficulty to be overcome to allow the clinician to arrive at a definitive diagnosis. It has been considered the existence both of common characteristics among some pathologies and the presence of univocal characteristics. The combination of all these features allowed us to give a presumptive name to the observed pathology, as every pathology will always have univocal features, but often they are not immediately detectable. We believe that our methodology can help to follow a method that includes, unknowingly, the differential diagnosis [19,20].

We underline that the algorithm has been realized using the clinical and histological features reported in specialized articles and books, but we do not exclude that in some subjects, the pathology may present a different clinical picture or that the patient may report information that is not completely accurate. In this case, the use of LinguApp could lead to an incorrect diagnosis; however, we believe that, for many patients, following this type of pathway can lead to a correct diagnosis in a quick and easy way. Therefore, we consider fundamental a meticulous anamnesis of the patient, in order to have detailed information on the evolution of the lesion, on the symptomatology of the same, and on the habits/risk factors of the patient, in order to be able to answer in an unequivocal way the questions posed in the algorithm [17,19].

Moreover, the use of the application over time will refine the type of questions, making them increasingly targeted. However, there is no doubt that the idea of being able to create sets of diseases and, within these, gradually find differences and then create subgroups, may be one of the correct methods to reach the recognition of the disease and then the diagnosis.

The proposed methodology is still in use today and provides a fast track to hospital admission for patients requiring direct intervention, without the need to have contact with any further people at risk of COVID-19 infection. This best practice could continue to be pursued after the contingent emergency as it generally simplifies the procedure from both the healthcare personnel and patient perspectives and it shortens the waiting time.

Finally, we point out that our application has been developed in flexible and logically separated modules. This would allow to quickly modify each single module to deal with different diagnostic problems, based on a proper questionnaire and on the submission of photos or videos. For example, LinguApp can be addressed to general practitioners, dentists, pediatricians, and dermatologists, presenting a more specific questionnaire for the collaboration of the various branch specialists. This is a strong point compared to other similar mobile applications currently available, specific to particular diseases.

So far, apps have been developed in various countries to track infected people (generally, on a voluntary basis) [24,25,26,27,28] or to make diagnoses through established approaches [29,30,31,32,33] or neural network-based techniques [34] that require extensive hardware resources. The Italian government has recently confirmed the sponsorship of remote diagnostics in all forms. Obviously, preference should be given to user-friendly tools that do not require special assets from the medical staff and especially from patients. From a general point of view, mobile technologies cannot replace a direct contact with the patient, but can contribute to the rapid diagnosis and maintain a high safety level for both operators and patients themselves, limiting their access to specialist facilities only in cases that are really necessary.

## Appendix A

The questionnaire in JSON format currently used by LinguApp is provided as Additional Material together with a short video to show how the Web and Mobile interfaces work. All these files can be downloaded at https://tinyurl.com/linguapp, accessed on 10 January 2022.

We introduce here a couple of main snippets. This codes refer to the actual questions that are proposed according to Figure 2.

When a path needs to be rendered, it is passed as state to Algorithm A1, which reads the key of each node in the list. Using this key, we can access the node’s label, if one is available, and the text of all its questions. A variant of this code is used to display, for all but the last node, only the selected alternative, instead of all of them.

**Algorithm 1** Current decision display.

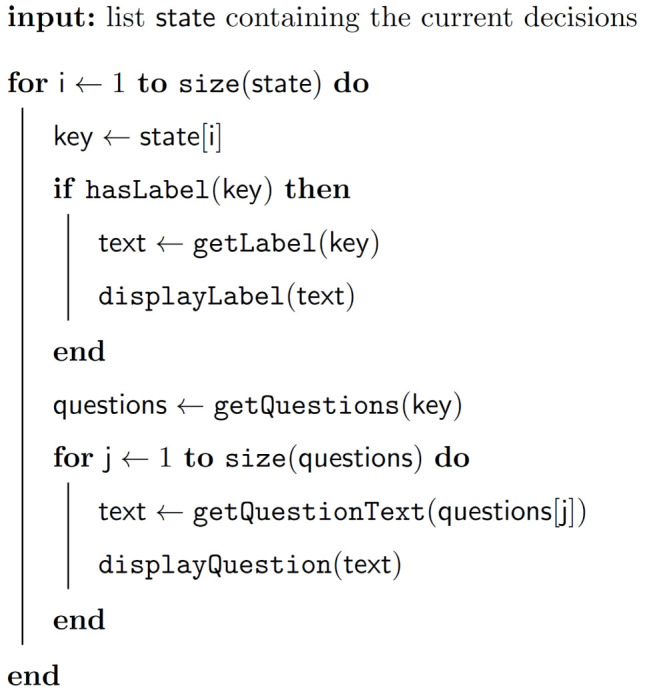

Algorithm A2 is executed when an alternative is selected, passing the index of the node involved in the stack as current and the index of the chosen alternative from the questions array as answer. This procedure removes the nodes in the path that come after current, which are only present if the patient is going back, and then adds the key of the node that is pointed to by the selected alternative.

**Algorithm 2** Decision stack update.
